# Perioperative tranexamic acid in abdominally based free-flap breast reconstruction: A single-surgeon retrospective series

**DOI:** 10.1016/j.jpra.2026.04.025

**Published:** 2026-04-30

**Authors:** Aaron I. Ahdoot, Jason C. Llaneras, Garrison A. Leach, Alvin T. Nguyen, Chris M. Reid

**Affiliations:** Department of Surgery, Division of Plastic Surgery, UC San Diego School of Medicine, La Jolla, CA, USA

**Keywords:** Tranexamic acid (TXA), Deep inferior epigastric perforator (DIEP) flap, Autologous breast reconstruction, Microvascular free-flap microsurgery, Hematoma, Venous thromboembolism (VTE)

## Abstract

**Background:**

Tranexamic acid reduces perioperative bleeding in many surgical settings, but its safety in microvascular breast reconstruction remains incompletely defined given theoretical concerns for anastomotic thrombosis. We evaluated outcomes after routine perioperative tranexamic acid in abdominally based free-flap breast reconstruction and contextualized findings with the evolving microsurgical literature.

**Materials/Patients and Methods:**

A retrospective review was performed of consecutive patients undergoing abdominally based deep inferior epigastric perforator free-flap breast reconstruction by a single surgeon after adoption of routine tranexamic acid (February–December 2025). All included patients received 1 g intravenous tranexamic acid at anesthesia induction and an additional 1 g at closing. Primary outcomes included hematoma, flap thrombosis, venous thromboembolism, and transfusion. Secondary outcomes included wound complications, inpatient complications, and length of stay.

**Results:**

Seventy-one reconstructions were performed in 46 patients (46 unilateral, 25 bilateral) with median follow-up of 6 months. Flap survival was 100% with no inpatient complications. No hematomas, flap thrombosis events, or transfusions occurred. Delayed complications occurred in 9 patients (19.6%), most commonly donor-site wound healing (8.7%) and skin-paddle healing (4.3%). Two patients (4.3%) required interventional radiology drainage for an abscess and seroma, and one patient experienced a segmental pulmonary embolism.

**Conclusions:**

In this single-surgeon series, perioperative tranexamic acid use during free flap breast reconstruction was safe and not associated with thrombotic or bleeding-related complications. Combined with the limited existing literature, these findings support tranexamic acid as a safe adjunct in microvascular breast reconstruction. Larger, multicenter prospective trials are needed to validate tranexamic acid’s role in this population.

## Introduction

Tranexamic acid (TXA) is an antifibrinolytic agent that inhibits plasminogen activation and fibrinolysis by binding to plasminogen and blocking its interaction with fibrin, thereby preventing dissolution of the fibrin clot. TXA has become a mainstay for reducing perioperative blood loss across a wide range of surgical specialties, demonstrating efficacy in reducing bleeding-related mortality without increasing thromboembolic risk.^1^

In plastic surgery, its use has expanded as evidence supports favorable hemostatic benefits without a clear increase in thromboembolic complications in many procedural settings.^2^, ^3^, ^4^ Multiple systematic reviews and meta-analyses have demonstrated that intravenous TXA significantly reduces blood loss and transfusion requirements in craniofacial surgery, cosmetic surgery, and burn care.^2^^,^^3^ In breast surgery specifically, multiple meta-analyses have demonstrated that perioperative TXA administration significantly reduces hematoma formation and seroma formation, with no thromboembolic events reported.^5^, ^6^, ^7^, ^8^, ^9^ Studies in mastectomy with immediate implant-based reconstruction have shown 60% decreased odds of hematoma formation with TXA use.^7^^,^^10^ However, the balance between improved bleeding control and potential microvascular risk is uniquely relevant in autologous free flap breast reconstruction, where flap perfusion depends on the patency of small-caliber anastomoses and postoperative hematoma can threaten both flap survival and aesthetic outcomes.

Despite growing adoption, the safety profile and clinical impact of perioperative TXA in microvascular breast reconstruction remain incompletely characterized, with relatively limited data specific to autologous free flap cohorts.^2^^,^^3^ The most comprehensive reviews acknowledge that further studies are required to provide quantitative conclusions on the effects of TXA administration in microsurgery.^2^ Accordingly, we present a single-surgeon experience evaluating perioperative TXA use in microvascular breast reconstruction and contextualize these findings through a focused review of the current literature on safety and postoperative outcomes in this population.

## Materials/Patients and methods

### Study design and cohort

A retrospective review was performed of all patients who underwent autologous breast reconstruction using abdominally based deep inferior epigastric perforator (DIEP) free flaps performed by the senior author (CMR) between February 2025 and December 2025, after adoption of routine perioperative TXA. Cases were identified from the surgeon’s operative log and corresponding medical records. Patients were included if they underwent abdominally based free-flap breast reconstruction during the study period; no exclusions were applied other than incomplete records precluding outcome assessment.

Given the consistently high baseline performance of the senior author’s practice prior to TXA adoption, including no documented flap losses and low rates of hematoma and transfusion, this study was designed as a descriptive safety analysis rather than a comparative cohort study. The primary aim was to evaluate perioperative outcomes following routine TXA implementation and assess for any signal of increased thrombotic or wound-related complications.

The study was approved by the University of California, San Diego Institutional Review Board (IRB #803099), and STROBE guidelines were followed.

### TXA protocol

All included patients received 1 g intravenous TXA at anesthesia induction and an additional 1 g at closing.

### Outcomes

Primary outcomes included postoperative hematoma, flap thrombosis, venous thromboembolism (VTE), and blood transfusion requirement. Secondary outcomes included wound complications (flap-related and donor-site), inpatient complications, and discharge timing, assessed via length of stay (days). Complications were assessed during the index hospitalization and throughout available follow-up. Descriptive statistics were used to summarize patient, operative, and outcome variables using python (version 3.13.5).

## Results

### Cohort characteristics

Seventy-one abdominally based free-flap breast reconstructions were performed in 46 patients between February 11, 2025 and December 30, 2025 (Table 1). Procedures included 46 unilateral and 25 bilateral reconstructions, with a median follow-up of 6 months. The median interval from mastectomy to flap reconstruction was 11 months (IQR, 0–43.3). Twenty-eight patients had a history of chest wall radiation, and 26 received neoadjuvant chemotherapy. Among bilateral reconstructions, 14 of 25 cases (56%) received intraoperative TXA re-dosing (Table 2).

**Table 1:** Demographic and outcomes data.

	Median [IQR]	n (%)
Number of patients		46
Age at flap operation, years (n = 46)	48.00 [41.25, 56.75]	
Sex (n = 46)		
Female		46 (100%)
Race (n = 46)		
White		34 (73.9%)
Other		8 (17.4%)
Unknown		2 (4.3%)
Asian		1 (2.2%)
Black		1 (2.2%)
Ethnicity (n = 46)		
Hispanic or Latino		28 (60.9%)
Not Hispanic or Latino		18 (39.1%)
BMI, kg/m² (n = 46)	28.55 [26.02, 30.88]	
Smoking history <10 years (n = 46)		3 (6.5%)
Current smoking (N = 46)		0 (0.0%)
Hypertension (N = 46)		5 (10.9%)
Diabetes mellitus (N = 46)		2 (4.3%)
Previous breast reconstruction/augmentation (N = 71)		21 (29.6%)
Delayed reconstruction (N = 71)		54 (76.1%)
History of chest wall radiation (N = 71)		28 (39.4%)
Neoadjuvant chemotherapy (N = 46)		26 (56.5%)

**Table 2:** Operative & perioperative characteristics.

Surgical Characteristics	Median [IQR]	n (%)
Delayed reconstruction	—	54 (76.1%)
Time from mastectomy to flap, months	11.00 [0.00, 43.25]	—
Number of reconstructions	—	71
Reconstruction laterality (recon-level)	—	—
└ Unilateral reconstructions	—	46 (64.8%)
└ Bilateral reconstructions	—	25 (35.2%)
Skin paddle used (N = 71)	—	44 (62.0%)
Length of stay, days	1.00 [1.00, 1.00]	—
Discharge timing	—	—
└ Discharge POD1	—	44 (95.7%)
└ Discharge POD2	—	2 (4.3%)
Follow-up duration, months (n = 46)	6.00 [2.00, 8.00]	—

### Postoperative outcomes and complications

Flap survival was 100%, with no flap failures recorded or inpatient complications. One flap-related issue occurred (2.2%), consisting of minor delayed wound healing at the skin paddle (Table 3).

**Table 3:** Surgical complication data.

Complication	n	%
**Microvascular thrombosis**	0	0.0%
**Flap loss**	0	0.0%
**Hematoma**	0	0.0%
**Transfusion**	0	0.0%
**Unplanned return to OR**	0	0.0%
**VTE (segmental PE)**	1	2.2%
**Delayed healing at donor site**	5	10.9%
**Skin paddle delayed wound healing**	2	4.3%
**Breast abscess requiring IR drainage**	1	2.2%
**Seroma requiring IR drainage**	1	2.2%
**Readmission for refractory nausea/vomiting**	1	2.2%

Delayed complications were documented in 9 patients (19.6%) The most common events were delayed donor-site wound healing (8.7%), including delayed abdominal donor-site healing (4.3%), minor delayed donor-site wound healing (2.2%), and delayed donor-site healing (2.2%). Delayed wound healing at the skin paddle occurred in 2 patients (4.3%). IR-managed drainage was required in 2 patients (4.3%): one for a left breast abscess (2.2%) and one for a seroma occurring in the setting of a segmental pulmonary embolism (2.2%). One patient was readmitted for refractory nausea/vomiting (2.2%). No hematomas, flap thrombosis events, or transfusions occurred.

### Comparative literature context (free-flap breast reconstruction with perioperative TXA use)

The literature review identified three additional Level III studies evaluating TXA use in free flap breast reconstruction, all of which reported low rates of flap-related thrombotic events and major bleeding complications (Table 4). Lardi et al. compared 63 TXA-treated flaps with 35 control flaps and reported no microvascular thrombosis in the TXA group versus one venous thrombosis (3%) in controls; hematomas occurred in both groups (TXA: 10%, control: 18.2%), with no DVT in either group.^11^ Leow et al. evaluated 150 TXA-treated patients (1 g before induction) versus 69 controls, reporting no flap failures and one venous thrombotic event in the TXA cohort, with no increase in complications compared with controls.^12^ Moursi et al. reported a prospective matched cohort of 96 TXA flaps versus 95 control flaps (TXA given as 1 g IV after microvascular anastomosis), observing one microvascular thrombosis in the TXA group with no flap failures or DVT, and no significant differences in bleeding or thrombotic outcomes compared with controls.^13^

**Table 4:** Overview of TXA use in free flap breast reconstruction.

Reference	Design	Level of Evidence	Flaps	Methods	Results	Conclusions
Lardi et al.^11^	Retrospective, single-surgeon cohort	III	Free flaps for breast reconstruction	TXA group: 50 patients (63 flaps) TXA. Titrated intraoperatively according to EBL (100 ml = 1 g, 200 ml = 2 g, 300 ml = 3 g) Control group; 33 patients (35 flaps) no TXA.	TXA group: no microvascular thrombosis, 5 hematomas (10%), 0 DVT, 2 flap losses (SGAP, DIEP) Control Group; 1 thrombosis of flap vein (3%), 6 hematomas (18.2%), 0 DVT.	TXA significantly reduced intraoperative blood loss and transfusion. No increase in thrombotic events.
Leow et al.^12^	Retrospective, single-center cohort	III	Free flaps for breast reconstruction	TXA group: 150 Patients received 1 g TXA prior to induction of anesthesia. Control group: 69; not given TXA during their hospital stay.	TXA group: 0 flap failures, 1 venous thrombotic event. No increase in complications vs control.	TXA was not associated with increased thrombotic or flap-related complications.
Moursi et al.^13^	Prospective, single-surgeon matched cohort	III	Free flaps for breast reconstruction	TXA group: 81 pateints (96 flaps) 1 g IV after microanastomosis Control group: 82 patients (95 flaps). No TXA during their hospital stay.	TXA group: 1 microvascular thrombosis in TXA group; no flap failure or DVT. No significant differences compared to control.	TXA use was not associated with increased thrombotic or bleeding complications. Only significant difference was LOS was shorter in the TXA group.
(current study)	Retrospective, single-surgeon cohort	III	Free flaps for breast reconstuction	71 flaps (46 patients) received 1 g IV TXA at induction and an additional 1 g at closing.	0 microvascular thrombosis, hematoma, or transfusions. 7 minor donor site wounds, 1 VTE. No major complications or flap losses.	TXA was safely administered without flap or thrombotic complications.

In the current single-surgeon cohort (71 DIEP flaps in 46 patients), outcomes were consistent with prior reports, with no hematomas, no microvascular thrombosis events, and no transfusions, and 100% flap survival.

## Discussion

This single-surgeon series of 71 abdominally based free-flap breast reconstructions demonstrates that perioperative TXA administration is associated with favorable safety outcomes in microvascular breast reconstruction, with no hematomas, flap thrombosis events, or transfusions recorded, and a 100% flap survival rate. Importantly, this study is not intended to demonstrate superiority of TXA over standard practice, but rather to evaluate whether its routine use introduces additional risk in a microsurgical population. These findings align with emerging evidence supporting the hemostatic benefits of TXA without increased microvascular or thromboembolic risk in autologous breast reconstruction.

The absence of flap failure and acute inpatient complications in this cohort supports the safety of TXA use in microvascular anastomoses. The theoretical concern that TXA might increase microvascular thrombosis risk has been addressed by several recent studies. Bengur et al. demonstrated in 397 head and neck free flap reconstructions that TXA reduced transfusion rates and blood loss without increasing flap vascular compromise or thromboembolic events.^14^ Similarly, a trauma study found no difference in flap complications or failure rates between patients receiving TXA for hemorrhage control versus those who did not.^15^ Lastly, Leow et al. performed a retrospective single-center cohort study of 240 patients undergoing autologous free flap tissue transfer, with 160 patients receiving 1 g intravenous TXA at induction and 80 controls receiving no TXA. No differences were found between the proportion of patients who developed microvascular thrombotic complications between the TXA and control groups.^12^ The 100% flap survival rate observed in this study compares favorably to published total flap failure rates of 1.2% to 2.7% in large studies.^16^, ^17^, ^18^, ^19^, ^20^, ^21^ Collectively, these findings support the hypothesis that TXA’s antifibrinolytic mechanism, which stabilizes physiologic clot formation rather than promoting pathologic thrombosis, does not adversely affect microvascular anastomoses.

Furthermore, the absence of hematoma formation in this cohort is particularly noteworthy given that hematoma represents one of the most common complications following DIEP flap reconstruction, with reported incidence rates ranging from 3.6% to 5.8% in large studies.^16^^,^^22^^,^^23^ Multiple meta-analyses in breast surgery with or without implant or free tissue reconstruction have shown that TXA significantly reduces the risk of hematoma formation, with odds ratios ranging from 0.33 to 0.48.^5^, ^6^, ^7^, ^8^, ^9^ In a cohort of patients only receiving mastectomy with delayed implant reconstruction, Weissler et al. reported a 60% reduction in hematoma rates with intravenous TXA without thromboembolic events.^10^ Our findings suggest that TXA use may confer the protective benefit against hematoma formation in DIEP free flap reconstruction without compromising microvascular outcomes, further supporting its use in microvascular breast reconstruction.

The 8.7% donor-site complication rate observed in this series, consisting entirely of delayed wound healing, compares favorably to published donor-site morbidity rates. Large systematic reviews report donor-site complication rates ranging from 5.9% to 10.9% for DIEP flaps, with hernia and bulge formation representing the most concerning long-term sequelae.^24^, ^25^, ^26^ The absence of hernia or bulge formation in this cohort may reflect meticulous fascial closure technique, though longer follow-up is necessary as these complications can manifest years after surgery.^27^ The 19.6% overall delayed complication rate, while notable, also falls within the expected range for autologous breast reconstruction. Bennett *et al*. reported overall complication rates of 32.9% at 2 years for all reconstruction types in the Mastectomy Reconstruction Outcomes Consortium study, with DIEP flaps demonstrating higher odds of any complication (OR 1.97).^28^

The potential impact of TXA on wound healing warrants consideration. Experimental studies have suggested that high doses of TXA may impair wound healing through effects on fibrinolysis and tissue remodeling.^29^ However, the dosing regimen used in this study (1 g at induction and 1 g at closure) is consistent with standard perioperative protocols and substantially lower than doses associated with these experimental findings. In our cohort, delayed wound healing occurred in 19.6% of patients, which falls within the expected range reported in the literature for DIEP flap breast reconstruction.^28^ Importantly, no clear increase in wound-related complications attributable to TXA was observed. Further prospective studies are needed to better define any potential relationship between TXA use and wound healing outcomes in microsurgical reconstruction.

The occurrence of one segmental pulmonary embolism (2.2%) in this cohort warrants careful consideration within the context of venous thromboembolism (VTE) risk in autologous breast reconstruction. The observed VTE rate falls well within the expected of 0.13% to 4% across large database studies, with most contemporary series reporting rates between 1.3% and 2.1%.^30^, ^31^, ^32^, ^33^, ^34^ Fischer et al. demonstrated that autologous reconstruction carries an odds ratio of 2.14 for VTE compared to mastectomy alone, with obesity significantly amplifying this risk.^30^ The single pulmonary embolism event in the present series occurred in a patient who also developed a seroma requiring drainage, suggesting that local wound complications rather than TXA administration may have contributed to the thrombotic event. Extensive systematic reviews and meta-analyses encompassing over 40,000 patients undergoing noncardiac surgery have consistently demonstrated that prophylactic intravenous TXA does not increase the risk of perioperative cardiovascular thromboembolic events, including pulmonary embolism and deep vein thrombosis.^35^, ^36^, ^37^, ^38^ In breast surgery specifically, multiple recent meta-analyses have reported zero thromboembolic events associated with TXA use.^6^^,^^39^^,^^40^

The present study has several limitations that warrant acknowledgment. The single-surgeon design, while ensuring technical consistency, limits generalizability across different surgical techniques and institutional protocols. While the absence of a control group limits the ability to directly compare outcomes with and without TXA, flap survival and perioperative outcomes in the senior author’s practice were consistently excellent prior to the adoption of TXA, including no documented flap losses and low complication rates. Following implementation of TXA, we similarly observed no meaningful change in these outcomes. Given this high-performing baseline, inclusion of a formal comparative cohort in this relatively small single-surgeon series would likely be underpowered and risk overinterpretation of non-significant differences. Accordingly, this study was designed as a safety-focused observational series, with findings interpreted in the context of existing literature that includes comparative cohorts. Lastly, the 56% re-dosing rate in bilateral cases suggests protocol variability that may have influenced outcomes. Despite these limitations, this series contributes to the growing body of evidence supporting the safety of perioperative TXA in microvascular breast reconstruction. In the specific context of breast reconstruction, where both hematoma formation and flap perfusion are critical determinants of success, TXA appears to offer hemostatic benefits without compromising microvascular outcomes. The 100% flap survival rate and absence of hematomas in this cohort, combined with a VTE rate that falls well within the expected range for this patient population, suggest that TXA can be safely incorporated into perioperative protocols for autologous breast reconstruction.

Future research should focus on prospective, randomized controlled trials comparing TXA to placebo in microvascular breast reconstruction, with adequate sample sizes to detect differences in rare but clinically significant outcomes such as flap failure and VTE. Standardization of TXA dosing protocols, including optimal timing, route of administration (intravenous versus topical versus combined), and duration of therapy, remains an important area for investigation. Long-term follow-up studies are needed to assess whether TXA influences late complications such as fat necrosis, flap volume retention, and donor-site hernia formation. Finally, cost-effectiveness analyses comparing TXA protocols to standard care, accounting for reductions in transfusion requirements, reoperation rates, and hospital length of stay, would provide valuable data for healthcare systems considering protocol implementation.

## Conclusion

In conclusion, this study demonstrates that perioperative TXA administration in abdominally based free-flap breast reconstruction is associated with excellent flap survival, absence of hematoma formation, suggesting hemostatic benefits without compromising microvascular outcomes. These findings combined with the limited existing literature provide additional evidence that the theoretical concerns regarding microvascular thrombosis may not translate to clinically significant risk when TXA is used judiciously in microsurgical breast reconstruction. While prospective randomized trials are needed to definitively establish TXA’s role in this setting, the current evidence suggests that TXA represents a promising adjunct for reducing bleeding-related complications without compromising flap outcomes or increasing thromboembolic risk in carefully selected patients undergoing autologous breast reconstruction.

## Declaration of AI and AI-assisted technologies in the writing process?

During the preparation of this work the authors used ChatGPT in order to improve text readability. After using this tool/service, the authors reviewed and edited the content as needed and take full responsibility for the content of the publication.

## Statement of ethical approval

This study was conducted in accordance with the ethical principles outlined in the World Medical Association Declaration of Helsinki (June 1964) and its subsequent amendments. Institutional review board approval was obtained from the University of California, San Diego (IRB #803099).

## Declaration of competing interest

The authors have no financial disclosures or conflicts of interests to report.
